# Extended curettage and heat ablation for desmoplastic fibroma of the distal femur with a 12-year follow-up period: A case report

**DOI:** 10.3892/ol.2014.2249

**Published:** 2014-06-12

**Authors:** MASAHIRO YOKOUCHI, YOSHINORI UENO, SATOSHI NAGANO, HIROFUMI SHIMADA, SHUNSUKE NAKAMURA, TAKAO SETOGUCHI, ICHIRO KAWAMURA, YASUHIRO ISHIDOU, SETSURO KOMIYA

**Affiliations:** Department of Orthopaedic Surgery, Graduate School of Medical and Dental Sciences, Kagoshima University, Kagoshima 890-8520, Japan

**Keywords:** desmoplastic fibroma, bone, curettage, heat ablation, bone graft

## Abstract

Desmoplastic fibroma is a particularly rare, benign but locally aggressive, primary bone tumor. Owing to previously published reports stating high recurrence rates following curettage, the recommended primary treatment for desmoplastic fibroma is a marginal to wide tumor resection. In the current report, the case of an athlete with desmoplastic fibroma of the distal femur who was treated with extended curettage, heat ablation and artificial bone grafting is described. The postoperative course was uneventful and no recurrence has been observed during the 12-year follow-up period. The patient is able to sit on his heels with a straight back, without pain and is able run a complete marathon.

## Introduction

Desmoplastic fibroma is a benign bone tumor, which is composed of spindle cells with minimal cytological atypia and abundant collagen production (1) that was initially described by Jaffe in 1958 (2). It is a particularly rare tumor with ~200 cases reported in the English literature to date (3,4). Desmoplastic fibromas are often locally progressive and aggressive (1). In cases where the tumors are located in the extremities, the recurrence rates following multi-modality treatments have been reported to be ≤55% (3). Although a standard treatment protocol for desmoplastic fibroma has not been established owing to the rarity of the disease, marginal or wide excision of tumors occurring in large bones is often advocated for the initial treatment due to the high recurrence rates following curettage. A recurrence rate of 55–72% has been reported following non-resection procedures, whereas tumor recurrence has been noted in only 17% of resection cases (1,3). Based on this data, previous case studies have indicated that, despite the greater functional loss, surgeons primarily opted for en-bloc or wide resection of the tumors followed by reconstruction, rather than curettage (4–7). In the present report, the case of a 26-year-old male athlete with desmoplastic fibroma of the distal femur is described. The tumor was treated by extended curettage with a high-speed burr, followed by heat ablation using a standard electrosurgical knife and reconstruction of the bony defect using artificial bone grafting. The patient was tumor free at the 12-year follow-up examination and exhibited excellent functional outcomes. These results indicate that desmoplastic fibromas occurring in the femur do not always require resection and that extended curettage with heat ablation may be an appropriate primary procedure for disease treatment.

## Case report

**Figure 1:**
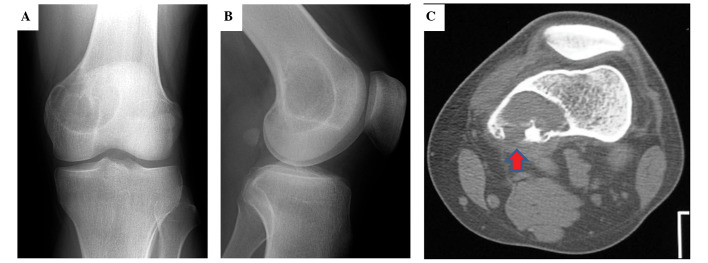
(A) Anteroposterior and (B) lateral radiographs of the distal femur tumor. An eccentric osteolytic lesion is visible in the medial condyle; however, there is no apparent periosteal reaction. (C) Axial computed tomography demonstrates a distinct lesion with (size, 48×45×30 mm) and destruction of the posterior cortex (red arrow).

**Figure 2:**
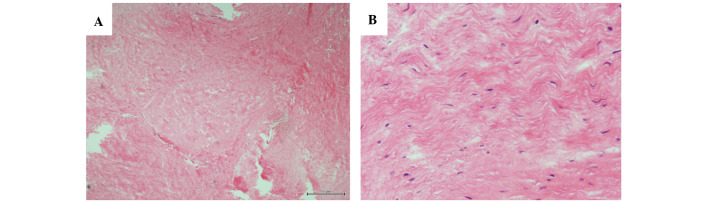
Microscopic examination demonstrates fascicles of spindle cells without cytological atypia separated by abundant collagen fibers. Increased cellularity and necrosis were not observed in the lesion, which also did not contain osteoids. Hematoxylin and eosin staining; magnification, (A) ×40 and (B) ×100.

**Figure 3:**
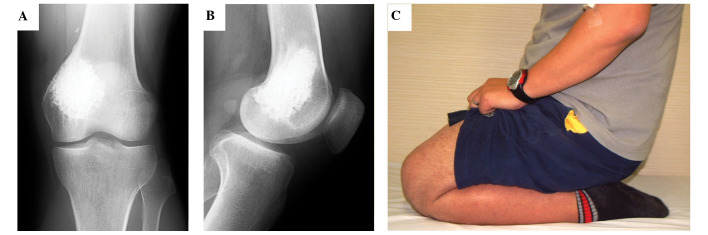
Repeated (A) anteroposterior and (B) lateral radiographs of the distal femur demonstrate no recurrence after the 12-year follow-up period. The radiolucent area between the implanted hydroxyapatite and the surrounding cancellous bone is completely absent. (C) Clinical range of motion of the knee; the patient is able to sit back on his heels with a straight back without experiencing pain.

A previously healthy 26-year-old male visited Kurauchi Orthopaedic Hospital (Miyazaki, Japan) for increasing pain in the left femur that began one year previously. Despite being a marathon runner, the patient had no history of trauma to this area. Results of a roentgenogram of the distal femur revealed an eccentric osteolytic lesion in the medial condyle. The patient was diagnosed with a benign bone tumor and was subsequently referred to the Department of Orthopaedic Surgery, Graduate School of Medical and Dental Sciences, Kagoshima University (Kagoshima, Japan) on May 21, 2001. During the first visit to our hospital, mild swelling with tenderness in the distal part of the thigh was observed. Results of laboratory examinations indicated that the white blood cell count, C-reactive protein level and erythrocyte sedimentation rate were normal. Physical examination revealed a normal range of motion in the knee joint. Radiographs demonstrated an eccentric osteolytic lesion with marginal sclerosis in the medial condyle. No periosteal reaction was observed (Figs. 1A and B). Computed tomography revealed a distinct lesion (mass size, 48×45×30 mm) and destruction of the posterior cortex (Fig. 1C). An open biopsy of the mass was performed and a pathological diagnosis indicated the presence of a desmoplastic fibroma. The patient did not consent to radical surgical treatment, such as en-bloc resection due to the potential extensive functional loss following surgery. Therefore, extended curettage with a high-speed burr, heat ablation with a standard electrosurgical knife and artificial bone grafting, using hydroxyapatite to fill the cavity, were performed. Macroscopic observation of the specimen revealed a yellow-white mass with a hard and rubber-like consistency. Histopathological analysis of the resected specimen verified the diagnosis of desmoplastic fibroma, which had been indicated by a previous biopsy (Fig. 2). The postoperative course was uneventful and no recurrence or metastasis was observed during the 12-year follow-up period (Fig. 3A and B). Currently, the patient’s functional outcome is excellent; the range of motion in the knee joint is not limited and the patient is able to sit on his heels with a straight back without experiencing pain (Fig. 3C). The patient is also able to run a complete marathon and, according to the Musculoskeletal Tumor Society Staging system, achieved a score of 100% (8).

Written informed consent was obtained from the patient for publication of this case report and the accompanying images.

## Discussion

Desmoplastic fibroma of the bone is a benign, non-metastatic, but locally aggressive tumor, which often occurs in adolescents and young adults. Long bones are the most common sites of occurrence with mandible, femur, pelvic, radial and tibial involvement in 23, 15, 13, 12 and 9% of cases, respectively (3). Therefore, when the disease involves the long bones in young patients, surgery is required to achieve good long-term functional outcomes without local recurrence following treatment. However, surgical experience is limited owing to the rarity of the disease; desmoplastic fibroma only accounts for ~0.1% of all primary bone tumors (9). As a result, surgical management of this rare condition remains controversial.

The primary surgical procedures for the treatment of desmoplastic fibroma include curettage, marginal or wide resection, cryosurgery and amputation (3). Böhm *et al* (3) analyzed 191 cases of desmoplastic fibroma of the bone and reported that recurrence rates following curettage, excision and wide resection were 55, 72 and 17%, respectively. Thus, it was concluded that the recommended primary treatment for desmoplastic fibroma of the bone is a marginal or wide resection of the tumors. Based on their analysis, the majority of physicians currently advocate en-bloc surgical excision with negative tumor margins. Several recent case studies have demonstrated that the majority of surgeons selected en-bloc or wide resection of tumors as the primary procedure when the tumors were located in the ulna (4), femur (5), ilium (6), scapula (7) and calcaneus (10). Therefore, desmoplastic fibroma tumors are predominantly treated with wide resection despite their lack of metastatic potential.

Extended curettage has been widely described in tumor surgery as a unique modality of surgical treatment for bone tumors. One of the advantages of this technique is its minimally invasive approach compared with conventional surgical procedures. Intralesional curettage has been considered the preferred treatment option for giant cell tumors (GCTs), another type of locally aggressive benign bone tumor, rather than resection, as it preserves the anatomy and function (11,12). Similar to desmoplastic fibroma, GCT is also noted for its potential to recur following curettage. Therefore, following thorough intralesional curettage, various adjuvant therapeutic procedures have been employed to enhance local control, such as liquid nitrogen, heat ablation, phenol treatment and acrylic cement. However, the effectiveness of such adjuvant therapeutic procedures during curettage remains unclear in cases of desmoplastic fibroma. In a study that included 191 cases (3), a high recurrence rate (55%) of desmoplastic fibroma was observed in patients who underwent curettage, however, it was not observed in those who underwent curettage with adjuvant therapy. According to their analysis, only four patients underwent curettage with adjuvant therapy and the recurrence rate in these patients was 25%, which was markedly lower compared with that in those who underwent curettage alone.

Recently, although certain reports have described the successful treatment of desmoplastic fibroma using curettage with lesion ablation (12), the follow-up duration in these studies was insufficient. Rastogi *et al* (10) proposed that when a resection causes major functional loss, an attempt at intralesional curettage is justified. In the present case, the patient was a young marathon runner and the lesion was located in the metaphysis of the femur. Therefore, heat ablation was selected as the adjuvant therapeutic procedure following thorough intralesional curettage in order to preserve good function of the knee and prevent local recurrence. While tumor resection is currently the preferred modality of treatment for desmoplastic fibroma, curettage with heat ablation has proved to be an acceptable alternative. Additional studies are required to investigate the effectiveness of adjuvant therapeutic procedures during surgery for the treatment of desmoplastic fibroma.

Furthermore, diagnosis of this rare disease is challenging and often inaccurate. Low-grade fibrosarcoma is often the most difficult differential diagnosis (14). Compared with desmoplastic fibroma, a typical fibrosarcoma is increasingly cellular with a herringbone pattern, which exhibits increased polymorphisms and higher mitotic activity (2). However, mitosis is not a prominent feature in low-grade bone fibrosarcoma (2). Therefore, in such cases, the diagnostic process is challenging and a final confirmation is often determined only after follow-up visits. In these instances, resection with negative margins is required to treat the tumor, rather than curettage.

In conclusion, the current report presents the rare case of a patient with desmoplastic fibroma of the distal femur who was successfully treated with extended curettage, heat ablation and artificial bone grafting. To preserve good, long-term function, extended curettage with heat ablation may be the preferred treatment for this benign lesion in the femur, rather than resection, owing to the lack of metastatic potential of the tumor.
